# 

**Published:** 1847-05

**Authors:** 


*To Readers and Correspondents.*—Communications are on hand, from Drs. Salisbury and Colegrove, which will appear in our next.

